# Enhancing High-Order Harmonic Generation Efficiency Through Molecular Size and Orientation Effects: A Pathway to Ultrafast Chemical Dynamics Studies

**DOI:** 10.3390/molecules30102133

**Published:** 2025-05-12

**Authors:** Shushan Zhou, Hao Wang, Dongming Yu, Nan Xu, Muhong Hu

**Affiliations:** School of Physics and Electronic Technology, Liaoning Normal University, Dalian 116029, China; zhoushushan@lnnu.edu.cn (S.Z.); 18018950396@163.com (H.W.); ydm747374@163.com (D.Y.); 13390283718@163.com (N.X.)

**Keywords:** high-order harmonic generation, ultrafast chemical dynamics, molecular alignment, time-dependent density functional theory

## Abstract

High-order harmonic generation provides a powerful tool for probing ultrafast chemical dynamics, such as electron transfer, bond breaking, and molecular structural changes, with attosecond temporal resolution. The strong laser fields used in HHG can also directly influence chemical reaction pathways and rates, enabling coherent control of reaction selectivity. However, enhancing the efficiency of harmonic emission remains a critical challenge in ultrafast science. In this study, we investigate the effects of molecular size and orientation on HHG efficiency using time-dependent density functional theory simulations. By analyzing the linear molecules $C_{18}H_{2}$, $C_{2}H_{2}$, and C_10_H_2_ under linearly polarized laser fields, we demonstrate that larger molecular sizes significantly enhance harmonic emission intensity. Our results reveal that $C_{18}H_{2}$, with its larger spatial dimensions, exhibits substantially higher harmonic intensity compared to smaller molecules like $C_{2}H_{2}$. This enhancement is further supported by examining charge redistribution and bond length changes during the HHG process. Additionally, we validate our findings with C_10_H_2_, a molecule of intermediate size, confirming the correlation between molecular size and harmonic efficiency.

## 1. Introduction

High-order harmonic generation (HHG) offers an ultrafast time-resolved capability to probe electron and nuclear dynamics in chemical reactions, such as electron transfer, bond breaking, and molecular structural changes [1,2,3]. Meanwhile, the intense laser fields driving HHG can directly influence reaction pathways and rates, even enabling selective control of reactions through coherent manipulation. This makes HHG a powerful tool for studying ultrafast chemical dynamics and molecular imaging [4]. Consequently, theoretical and experimental research on HHG emission has remained a crucial topic in the field of ultrafast chemical dynamics.

HHG refers to the generation of harmonics with frequencies hundreds or even thousands of times higher than the incident laser [5,6,7,8,9,10]. When atoms or molecules interact with strong laser fields, they emit high-energy photons, resulting in HHG. The underlying mechanism can be described by the semi-classical three-step model proposed by Corkum in 1993 [11,12]: first, the incident laser induces ionization of electrons from the target material; then, the liberated electrons accelerate under the influence of the laser field; finally, as the laser field reverses, the electrons return to the parent ion, recombining and emitting high-order harmonic photons.

Enhancing harmonic emission intensity enables the generation of more powerful attosecond pulses [13,14,15,16], providing a valuable tool for time-resolved spectroscopy in ultrafast chemical reactions. At the same time, stronger harmonic signals significantly improve the resolution and accuracy of molecular orbital imaging [4,17,18,19,20,21], while high-intensity harmonics create opportunities for conducting more complex chemical reaction experiments. To boost HHG intensity, extensive efforts have been made in both theoretical and experimental studies.

In 2017, Li et al. significantly enhanced the yield of the second plateau in solid-state high-order harmonic generation using a two-color superimposed field technique [22]. By adjusting the laser field parameters, they theoretically achieved the synthesis of a single attosecond pulse. Subsequently, in 2018, Liu et al. combined nanofabrication techniques with ultrafast laser technology, utilizing all-dielectric metasurfaces to enhance laser intensity and increase the damage threshold of the medium [23]. This approach led to an improvement in harmonic efficiency by nearly two orders of magnitude. In the same year, Franz et al. discovered that nanocone waveguides could significantly enhance HHG efficiency in semiconductor materials [24]. Additionally, research by Yao et al. demonstrated that under laser excitation with wavelengths exceeding 200 nm, the HHG intensity in bilayer MoS_2_ with a T-stacked structure exhibited a strong wavelength-dependent enhancement [25]. This finding provided new insights into improving solid-state HHG efficiency.

Beyond laser parameters and solid-state targets, molecular orientation effects also play a crucial role in determining HHG intensity. In 2005, Bandrauk et al. found that in short-cycle pulse fields, electron ionization efficiency was significantly higher when the carrier-envelope phase was anti-parallel to the permanent dipole moment of a nonpolar molecule compared to the parallel configuration. They predicted that this phenomenon exists in all nonpolar molecules [26]. Li et al. analyzed the HHG emitted by CO_2_ molecules at different orientation angles in strong laser fields and observed that the vertical harmonic efficiency was comparable to the parallel harmonic efficiency at small orientation angles but significantly lower at larger angles. Their study revealed a strong correlation between the vertical HHG efficiency of CO_2_ and its molecular structure [27]. In 2017, Shi et al. investigated the HHG spectra of asymmetric molecules in linearly polarized laser fields and proposed a novel method to determine molecular orientation by calibrating HHG yield from highly polar molecules [28].

In addition to orientation effects, molecular size also significantly impacts HHG intensity. In 2007, the study by Takahashi et al. demonstrated that by introducing booster harmonics generated from Xe atoms in a mixed gas of Xe and He, the high-order harmonic yield from He can be significantly enhanced, with an increase of up to 4000 times [29]. In 2010, Hu et al. analyzed the collision probability distributions of H_2_, D_2_, and T_2_ isotopic molecules in strong fields and found that, under identical conditions, heavier isotopes exhibited higher HHG yields [30]. In 2020, Romanov et al. discovered that atomic-scale polarization potential structures could dramatically enhance HHG efficiency under high-intensity fields [31].

Based on these findings, we considered molecular size effects and selected larger molecules to enhance HHG intensity through specific molecular orientations in linearly polarized laser fields. Using time-dependent density functional theory (TDDFT) [32,33,34] simulations, we investigated the HHG generated by $C_{18}H_{2}$, $C_{2}H_{2}$, and C_10_H_2_ molecules oriented perpendicularly to the polarization direction of the laser field. Our results show that under high-intensity laser fields, the HHG intensity of the larger $C_{18}H_{2}$ molecule is significantly higher than that of the smaller $C_{2}H_{2}$ molecule. By analyzing charge redistribution and bond length variations during the interaction process, we demonstrated that larger molecular sizes contribute to enhanced HHG intensity. The study of C_10_H_2_, an intermediate-sized molecule between $C_{18}H_{2}$ and $C_{2}H_{2}$, further validated this conclusion. This approach offers new possibilities for optimizing HHG emission efficiency.

## 2. Results and Discussion

**Figure 1:**
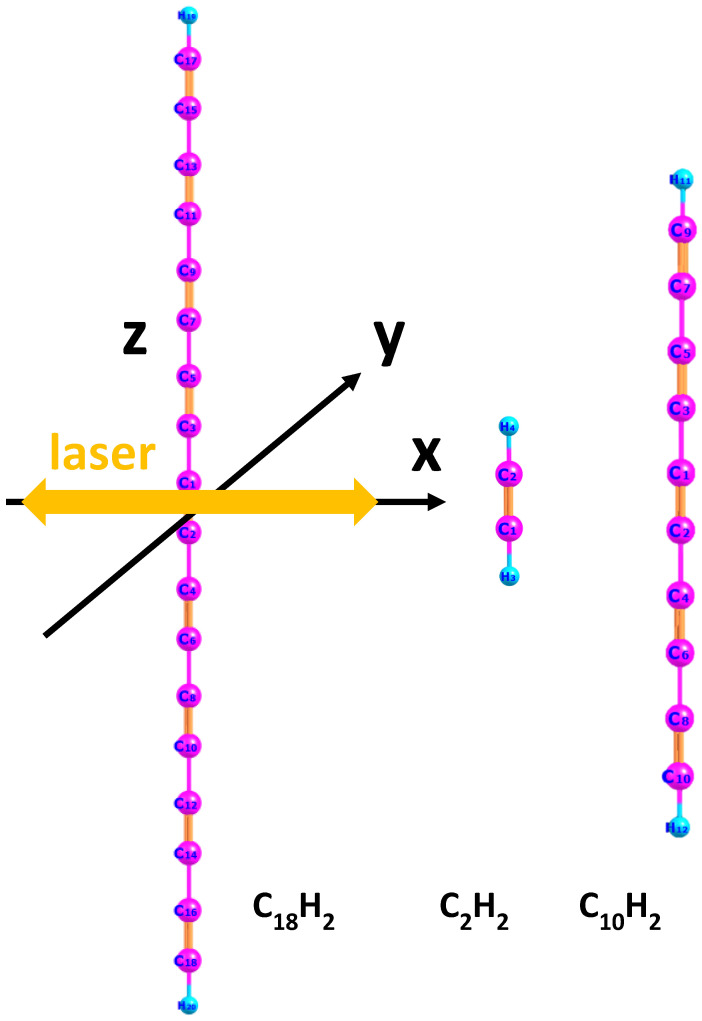
Schematic diagram of the molecular orientation of $C_{18}H_{2}$, $C_{2}H_{2}$, and C_10_H_2_ molecules with respect to the linearly polarized electric field. The spatial dimensions of the three molecules are 45.18 a.u. for $C_{18}H_{2}$, 6.25 a.u. for $C_{2}H_{2}$, and 25.7 a.u. for C_10_H_2_. The yellow arrow indicates the laser polarization direction, while the black arrows represent the x- and y- axis of the coordinate system.

First, we employed the DFT method using the open-source software Octopus (version 9.2) [35,36] to numerically simulate the ground-state properties of three molecules ($C_{18}H_{2}$, $C_{2}H_{2}$, and $C_{10}H_{2}$) under time-independent conditions, such as ionization potential and the wavefunction of the highest occupied molecular orbital (HOMO). The lengths of the molecular axes for the three molecules are 45.18 a.u., 6.25 a.u., and 25.7 a.u., respectively. We performed real-time and real-space computational simulations. The simulation box was in the shape of a parallelepiped, with half-lengths of 100 a.u., 40 a.u., and 100 a.u. along the three spatial directions. The grid spacing in the simulation box was set to 0.4 a.u. The calculated ionization potentials (HOMO eigenvalues) of the three molecules are 0.254 a.u., 0.456 a.u., and 0.294 a.u., respectively. The molecular axis is aligned along the z-axis of the Cartesian coordinate system. The relationship between the molecular orientation and the polarization direction of the linearly polarized electric field is illustrated in Figure 1. The yellow double arrow indicates the polarization direction of the laser field.

**Figure 2:**
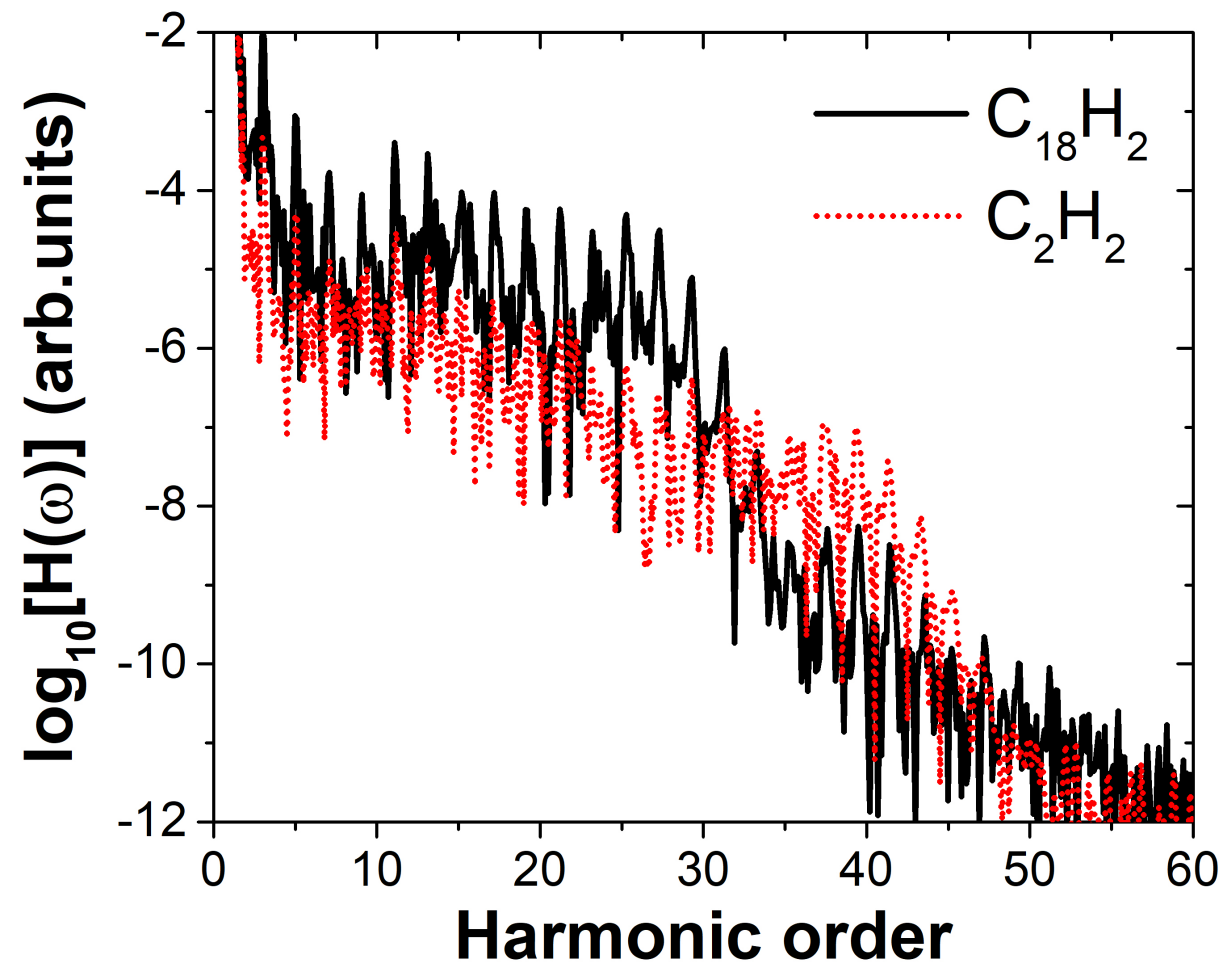
High–order harmonic generation spectra of the $C_{18}H_{2}$ and $C_{2}H_{2}$ molecules in the laser field. The relationship between the laser polarization direction and molecular orientation is shown in Figure 1. The linearly polarized electric field amplitude for C_18_H_2_ is 0.06 a.u. Under the same Keldysh parameter, the corresponding field amplitude for C_2_H_2_ is 0.079 a.u., with a wavelength of 800 nm for both. The laser envelope is trapezoidal, with both the rising and falling edges lasting one optical cycle and a plateau of four optical cycles.

**Figure 3:**
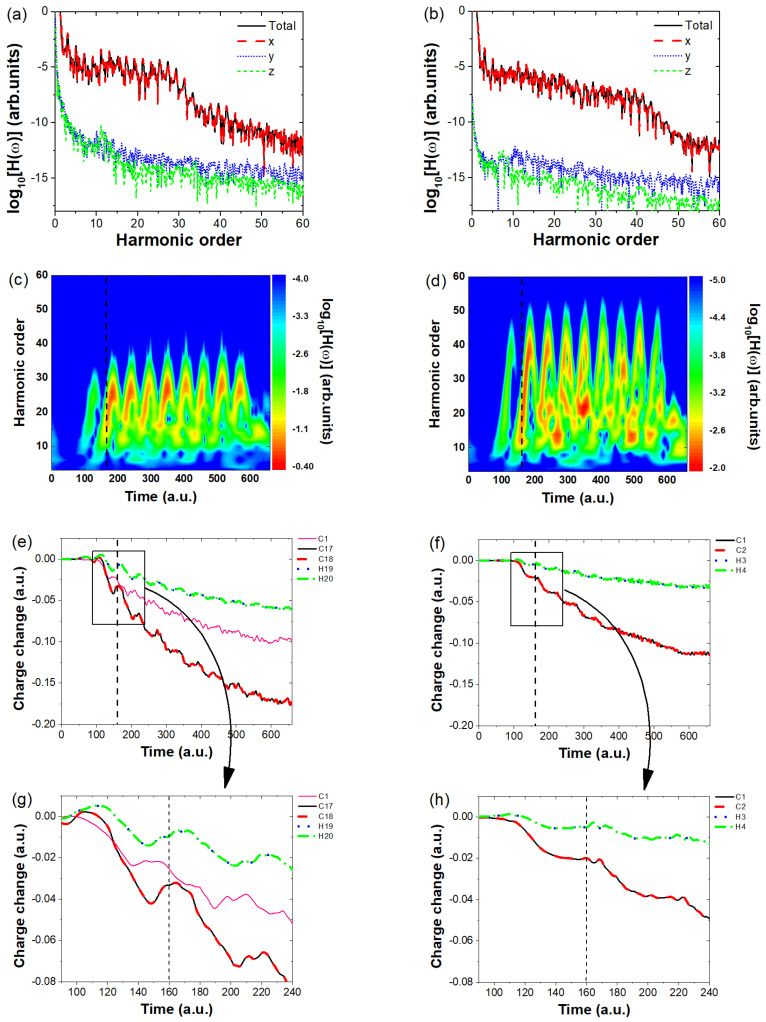
(**a**,**b**) show the overall harmonic spectra of $C_{18}H_{2}$ and $C_{2}H_{2}$, along with their component spectra for each direction. (**c**,**d**) present the time–frequency analysis of the harmonic spectra in the x- direction for $C_{18}H_{2}$ and $C_{2}H_{2}$, with the dashed lines indicating key harmonic emission moments. (**e**,**f**) illustrate the time evolution of the charge distribution around the atomic nuclei of $C_{18}H_{2}$ and $C_{2}H_{2}$, with the dashed lines highlighting the moments of harmonic emission. Here, the charge variation curves of C1–C16 are similar, only the curve for C1 is shown as a representative for clarity. To clearly observe the variations in the curves within the black solid boxes in (**e**,**f**), we have magnified these regions and presented them in (**g**,**h**), respectively, as indicated by the arrows on the curves.

We performed numerical simulations of the molecular harmonic emission under a linearly polarized electric field using the TDDFT method. The size of the simulation box is the same as that used in the time-independent calculations, with a time step of 0.08 a.u. Since the harmonic intensity depends on both the ionization and recombination probabilities, and the three molecules have different ionization potentials, we aimed to minimize the influence of varying ionization potentials on our conclusions. To achieve this, we simulated the harmonic generation processes of the molecules under different laser field intensities while keeping the Keldysh parameter γ constant [37]. This approach allows for a fair comparison of the resulting harmonic spectra under the same ionization regime. The Keldysh parameter $\gamma =\frac{\omega \sqrt{2I_{p}}}{E}$, where ω is the laser angular frequency, $I_{p}$ is the ionization potential and *E* is the electric field amplitude. We applied an electric field with an amplitude of 0.06 a.u. to the C_18_H_2_ molecule. Using the ionization potential of this molecule, we calculated the corresponding Keldysh parameter as γ=0.683. Based on this value of γ, the electric field amplitude for C_2_H_2_ was determined to be 0.079 a.u. Under identical laser parameters except for the field amplitude, the high-harmonic spectra of the two molecules were calculated, as shown in Figure 2. The black solid line represents the harmonic spectrum of $C_{18}H_{2}$, and the red dashed line represents the spectrum of $C_{2}H_{2}$. The relationship between the laser polarization direction and molecular orientation is shown in Figure 1. The linearly polarized laser field has a wavelength of 800 nm. The field envelope is trapezoidal, with both the rising and falling edges lasting one optical cycle, and the plateau spans four optical cycles. As shown in Figure 2, the high-harmonic spectra of C_18_H_2_ and C_2_H_2_ both exhibit the typical “decreasing–plateau–cutoff” structure, characteristic of HHG spectra. It can be observed that the cutoff positions appear near the 25th and 39th harmonic orders, respectively. This difference in cutoff positions originates from the different ionization potentials of the two molecules. According to the cutoff law [38] $N=(I_{p}+3.17U_{p})/\omega$, where $U_{p}=E^{2}/\left(4\omega^{2}\right)$ is the ponderomotive energy, the calculated cutoff orders are 20 and 34, respectively. The discrepancy arises from the use of the HOMO ionization potential $I_{p}$ in the calculation. However, since both molecules possess multi-orbital structures and the HOMO ionization potentials are relatively small, the actual cutoff observed in the spectra corresponds more closely to inner orbital ionization potentials. Nevertheless, our main focus here is the difference in harmonic intensity between the two molecules. As shown in the figure, in the range of the 3rd to 20th harmonic orders, the intensity of C_18_H_2_ is about one order of magnitude higher than that of C_2_H_2_. In the high-energy region (23rd–27th orders), the intensity difference reaches approximately two orders of magnitude. To investigate the reason why the harmonic intensity of $C_{18}H_{2}$ is higher than that of $C_{2}H_{2}$, we conducted a systematic study of the harmonic emission from both molecules. The results are presented in Figure 3.

We first investigate which directional harmonic emission contributes to the overall enhancement in harmonic intensity. To this end, we plot the harmonic intensities along the x-, y-, and z-directions of polarization for both $C_{18}H_{2}$ and $C_{2}H_{2}$ and compare them with the total harmonic intensity, as shown in Figure 3a,b. From these figures, we observe that for both molecules, only the x-direction harmonic intensity overlaps with the total harmonic intensity, whereas the y- and z-direction intensities are significantly lower (by approximately 6 orders of magnitude in plateau region) and can thus be neglected. This confirms that the overall harmonic intensity in both cases originates solely from the x-direction emission. Since the total harmonic intensity is determined by the harmonic emission along the laser polarization direction, the key question is: what causes the significant difference in their harmonic strengths? To answer this, we analyze the charge variation around the atomic nuclei of both molecules. First, we perform a time-frequency analysis to identify the primary moments of harmonic emission. The results are shown in Figure 3c,d, where the horizontal axis represents the time of harmonic emission, the vertical axis denotes the harmonic order, and the color scale indicates the harmonic intensity. Multiple bright stripes in both figures suggest that harmonic emission occurs at several key moments, such as t=160 a.u., 220 a.u., and 280 a.u. To further investigate the differences in harmonic strength, we take t=160 a.u. as an example and analyze the charge variation around the atomic nuclei of both molecules. The calculated charge evolution over time is presented in Figure 3e,f, with dashed lines marking the harmonic emission moments. As seen in Figure 3e, the horizontal axis represents different time moments, and the vertical axis shows the variation in the charge around different nuclei at each time compared to the charge at *t* = 0. At t=160 a.u., only the charge variation near the 17th and 18th carbon atoms shows an increasing trend, which proves that the harmonic emission at this moment is mainly due to electron recombination near the C17 and C18 nuclei, leading to the harmonic emission. To clearly observe the changes in the electronic charge around different nuclei near the time indicated by the dashed lines, we have magnified the region within the black solid box and presented it in Figure 3g, as indicated by the arrows on the curves (a similar approach is also applied in Figure 3f,h). At time t=150a.u., the charge around the C17 and C18 nuclei increases from approximately −0.04 to around −0.03, indicating a noticeable increase in the number of returning electrons. In contrast, for the C_2_H_2_ molecule, the charge around the C1 and C2 nuclei remains steady at about −0.02, showing no significant increase. After t=160a.u., the electron population continues to decrease. Here, the charge variation curves of C1–C16 are similar; for clarity, only the curve of C1 is shown as a representative in Figure 3c. The overall charge variation around these two nuclei is much more pronounced compared to the rest, highlighting the strong influence of molecular size on the electron dynamics in $C_{18}H_{2}$. For $C_{18}H_{2}$, the total charge near the nuclei generally decreases over time; however, at certain instants (such as t=160 a.u.) a noticeable but small increase is observed. This indicates the occurrence of electron recombination with the nucleus, leading to the emission of high-order harmonics. In contrast, for $C_{2}H_{2}$ (Figure 3f,g), the charge variations around the two nuclei (C1, C2) are nearly identical, with their curves almost overlapping, and the overall variation remains small. In contrast, the charge variation around the H3 and H4 nuclei is minimal, indicating that their contribution to the harmonic emission is negligible. Moreover, at multiple recollision moments, the increase in charge amount is not significant, indicating that fewer electrons recollide with the atomic nucleus, resulting in very low harmonic emission intensity.

**Figure 4:**
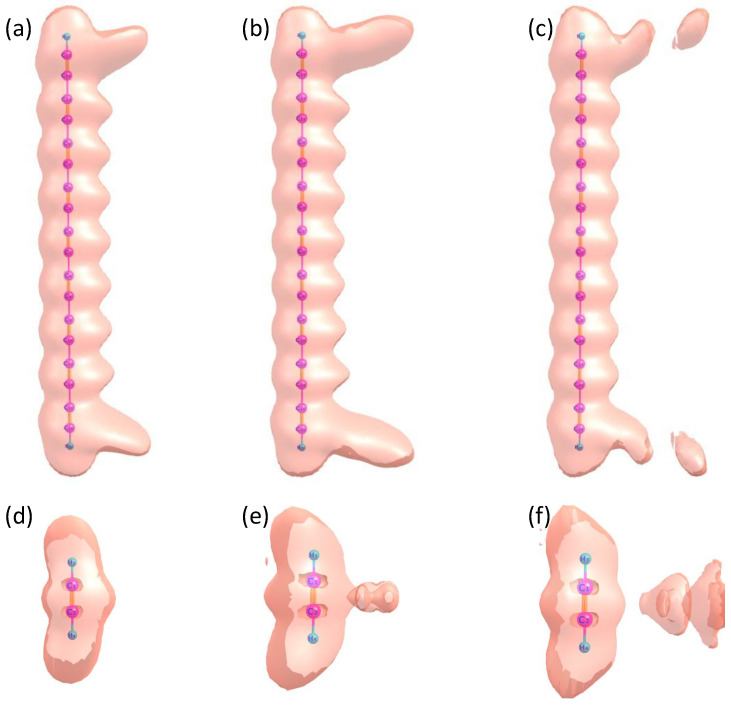
Time–dependent electron localization function (TDELF) of $C_{18}H_{2}$ and $C_{2}H_{2}$ under the influence of a linearly polarized laser field. The electric field parameters for the two cases are taken from those shown in Figure 2. (**a**–**c**) show the ELF of $C_{18}H_{2}$ at t=160 a.u., t=170 a.u., and t=180 a.u., respectively. (**d**–**f**) present the ELF of $C_{2}H_{2}$ at the same time instants: t=160 a.u., t=170 a.u., and t=180 a.u.

To better visualize the distinct harmonic emission processes of $C_{18}H_{2}$ and $C_{2}H_{2}$ and to gain deeper insight into electron dynamics, we present the time-dependent electron localization function (ELF) of both molecules under a linearly polarized laser field in Figure 4. The electric field parameters for the two cases are taken from those shown in Figure 2. Figure 4a–c correspond to $C_{18}H_{2}$ at t=160 a.u., t=170 a.u., and t=180 a.u., respectively, while Figure 4d–f show the ELF of $C_{2}H_{2}$ at the same time instants. From Figure 4a–c, it is evident that at t=160 a.u., a small fraction of electrons in $C_{18}H_{2}$ recombine, leading to harmonic emission. The most significant charge redistribution occurs at both ends of the molecule, particularly around C17 and C18. From t=160a.u. to t=170a.u., the chemical bonds at the molecular terminals gradually break, eventually resulting in electron ionization. A similar phenomenon is observed in the ELF of $C_{2}H_{2}$. However, due to the significantly smaller molecular size of $C_{2}H_{2}$ compared to $C_{18}H_{2}$, no pronounced charge redistribution occurs at the molecular terminals. Instead, bond breaking primarily takes place in the central region between the two carbon nucleus, leading to electron ionization. This behavior aligns with the conventional harmonic emission mechanism and explains the substantially lower harmonic intensity of $C_{2}H_{2}$ compared to $C_{18}H_{2}$.

**Figure 5:**
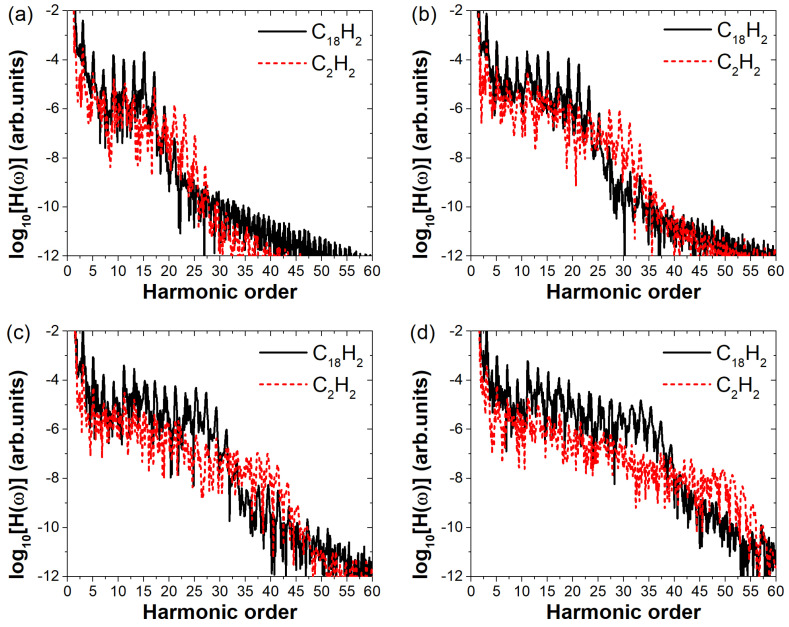
High−order harmonic spectra of $C_{18}H_{2}$ and $C_{2}H_{2}$ under linearly polarized laser fields of varying intensities. In Figures (**a**–**d**), the electric field amplitudes for C_18_H_2_ are 0.04 a.u. to 0.07 a.u., respectively. Under the same Keldysh parameter, the corresponding field amplitudes for C_2_H_2_ are 0.053 a.u., 0.066 a.u., 0.079 a.u., and 0.092 a.u., respectively.

To investigate the generality of the enhanced harmonic emission in $C_{18}H_{2}$ due to its larger molecular size, we systematically varied the laser field amplitude and computed the high-order harmonic spectra of $C_{18}H_{2}$ under different field strengths ranging from 0.04 a.u. to 0.07 a.u., as shown in Figure 5a–d, respectively. For comparison, we also calculated the harmonic spectra of $C_{2}H_{2}$. To avoid the influence of the ionization potential on the conclusions, we also perform comparisons under the condition that the Keldysh parameter γ remains equal. The four electric field amplitudes correspond to γ values of 1.025, 0.820, 0.683, and 0.586, respectively. By substituting these four sets of parameters into the Keldysh parameter formula, the electric field amplitudes used for simulating C_2_H_2_ should be 0.053 a.u., 0.066 a.u., 0.079 a.u., and 0.092 a.u., respectively. As shown in Figure 5, the harmonic cutoff positions of $C_{2}H_{2}$ are consistently higher than those of $C_{18}H_{2}$ in all four panels. This is attributed to the difference in their ionization potentials. Our primary focus, however, is on the difference in harmonic intensities between the two molecules. It is visually apparent from all four panels that $C_{18}H_{2}$ exhibits significantly higher harmonic intensities than $C_{2}H_{2}$. As shown in Figure 5a, at a relatively low field strength, the harmonic spectra of $C_{18}H_{2}$ and $C_{2}H_{2}$ exhibit minimal differences. Although $C_{18}H_{2}$ shows clearly higher intensity in the range of harmonic orders 1–17, only the 15th harmonic is about two orders of magnitude stronger than that of $C_{2}H_{2}$. This indicates that at low field strengths, the larger molecular size of $C_{18}H_{2}$ does not yet lead to a substantial enhancement in harmonic emission. However, as the electric field strength increases, as shown in Figure 5b–d, all harmonic orders in the plateau region of $C_{18}H_{2}$ become noticeably stronger than those of $C_{2}H_{2}$. Moreover, the intensity difference between the two molecules grows progressively with increasing field strength. In Figure 5d, the harmonic intensities from the 11th to the 35th order of $C_{18}H_{2}$ are approximately two orders of magnitude higher than those of $C_{2}H_{2}$. This demonstrates that the effect of molecular size on harmonic intensity becomes more pronounced with increasing electric field amplitude of the incident laser.

**Figure 6:**
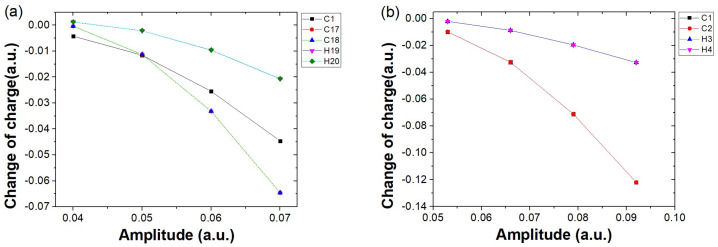
Charge variation around different atomic nuclei of (**a**) $C_{18}H_{2}$ and (**b**) $C_{2}H_{2}$ at the key harmonic emission time t=160 a.u., relative to their ground−state values. The horizontal axis represents different electric field amplitudes, while the vertical axis indicates the charge variation around the nuclei. The range of field amplitudes for both cases remains consistent with those in Figure 5a–d. Here, the charge variation curves of C1−C16 in the C_18_H_2_ molecule are similar; therefore, only the curve for C1 is shown as a representative for clarity.

To better understand how increasing electric field strength leads to a growing disparity in harmonic intensity between $C_{18}H_{2}$ and $C_{2}H_{2}$, we analyze the charge variation around different atomic nuclei at the key harmonic emission time t=160 a.u. under various field strengths. Since the charge variation around the H atoms in both molecules is small at all time moments, their contribution to the harmonic emission is negligible. Therefore, in Figure 6, we analyze only the variation of the charge around the carbon nuclei at the moment of harmonic emission as a function of the field strength. As shown in Figure 6a, the charge variation trends around the nuclei of C17 and C18, represented by red circles and blue triangles respectively, are nearly identical. The amount of charge variation for both increases with the field strength. A similar trend is observed for the nucleus of C1, represented by black squares. Since the charge variation trends around the other carbon nuclei (excluding C17 and C18) are consistent, C1 is used as a representative. At low field strength (E=0.04 a.u.), the charge variation around C17 and C18 is smaller than that around C1 and only slightly more significant than that around the hydrogen nuclei. At this point, electron localization around the terminal carbon atoms is not prominent, thus having little influence on the harmonic intensity, corresponding to the case shown in Figure 5a. As the field strength increases (the corresponding cases in Figure 5b–d), the charge variation around the terminal carbon nuclei C17 and C18 becomes more significant, clearly exceeding that around the hydrogen nuclei. Even at field strengths of 0.06 a.u. and 0.07 a.u. (corresponding to Figure 5c,d), the charge localization around C17 and C18 is clearly more pronounced than that around the other carbon nuclei represented by C1, highlighting the importance of electron recombination near the terminal carbon atoms in long-chain molecules for high harmonic generation. In contrast, for the case of $C_{2}H_{2}$, the behavior is much simpler: the charge variations around the two carbon nuclei, C1 and C2, remain consistent, indicating that there is no HHG mechanism similar to that observed in $C_{18}H_{2}$.

**Figure 7:**
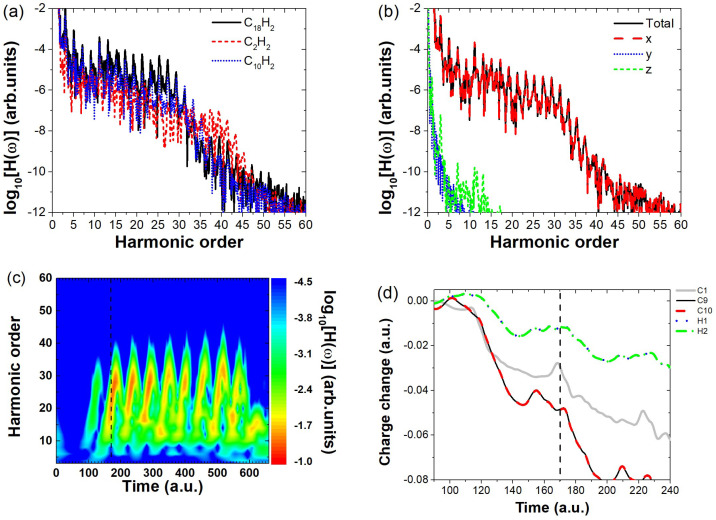
(**a**) High–order harmonic spectrum of C_10_H_2_ calculated under the same Keldysh parameters as in Figure 2, compared with $C_{18}H_{2}$ and $C_{2}H_{2}$. (**b**) The total high–order harmonic spectrum of the C_10_H_2_ molecule, as well as its harmonic components along the x-, y-, and z- directions. (**c**) Time–frequency analysis of the HHG spectrum for C_10_H_2_, where the dashed line marks a key harmonic emission time (*t* = 170 a.u.). (**d**) Evolution of charge variation around individual nuclei in C_10_H_2_ at the main harmonic emission time. Here, the charge variation curves of C1–C8 are similar, only the curve for C1 is shown as a representative for clarity.

To verify the generality of the above conclusions, we selected C_10_H_2_, whose spatial dimension lies between $C_{18}H_{2}$ and $C_{2}H_{2}$, as the target molecule. The HHG spectrum of C_10_H_2_ was simulated under the same field parameters as in Figure 2 (To use the same Keldysh parameters as in Figure 2, the electric field amplitude is set to 0.064 a.u.), and the results are shown in Figure 7a, where the blue dotted line represents the HHG spectrum of C_10_H_2_. The results indicate that, at this field strength, the harmonic intensity of C_10_H_2_ in the high-energy region (15th–27th order) falls between that of $C_{18}H_{2}$ and $C_{2}H_{2}$. Further analysis of the harmonic components along the x, y, and z directions (Figure 7b) reveals that the x-directional harmonic intensity is nearly identical to the total harmonic intensity, indicating that harmonic emission is primarily concentrated along the laser polarization direction. Additionally, time-frequency analysis (Figure 7c) shows that t=170a.u. is a key moment of HHG emission in C_10_H_2_. Examining the charge variation around different atomic nuclei around this moment (Figure 7d), We find that, the most significant charge variation occurs at the C9 and C10 nuclei (black solid line and red dashed line), where obvious electronic localization behavior is observed. In contrast, this is not the case for other carbon nuclei represented by C1 (gray thin solid line), as the charge variation around those nuclei generally shows a decreasing trend. Nor does it occur at the terminal H11 and H12 nuclei (blue dotted line and green dash-dotted line), since the charge variations near the hydrogen nuclei are minimal. These findings indicate that, similar to the $C_{18}H_{2}$ molecule, the localized electrons near the terminal carbon nuclei of the long-chain $C_{10}H_{2}$ molecule play a dominant role in harmonic emission. Consequently, the effective interaction length of $C_{10}H_{2}$ can be reasonably approximated by the length of its carbon backbone. These results further reinforce our previous conclusion: when the polarization direction of the linearly polarized electric field is perpendicular to the molecular axis, the spatial extent of a long-chain molecule has a significant impact on the HHG intensity. Specifically, as the field strength increases, larger molecules tend to exhibit stronger harmonic emissions.

## 3. Theory and Method

The TDDFT method has proven to be a powerful tool for investigating molecular high-order harmonic generation in intense laser fields [39,40,41]. Notably, its application to Xylene molecules [42,43,44] and complex solid-state systems [45,46] has demonstrated remarkable consistency with experimental observations.

Based on the Runge–Gross theorem [47], the time-dependent external potential uniquely determines the time-dependent single-electron density for a many-body system evolving from a specified initial state. Under the length gauge and dipole approximation, the electron dynamics in molecular systems subjected to linearly polarized laser pulses are governed by the time-dependent Kohn–Sham (KS) equations for orbitals $\psi_{i}\left(r,t\right)$, expressed as follows (atomic units are used throughout):

(1) $$\begin{array}{c} i\frac{\partial }{\partial t}\psi_{i}\left(r,t\right)=\left[-\frac{1}{2}\nabla^{2}+V_{\mathrm{KS}}\left[\rho \right]\left(r,t\right)\right]\psi_{i}\left(r,t\right),\;i=1,\ldots N. \end{array}$$

In this formulation, *i* denotes the orbital index, while *N* represents the total number of Kohn–Sham orbitals. The time-dependent electron density, ρ(r,t), is expressed as:

(2) $$\begin{array}{c} \rho \left(r,t\right)=2\sum_{j=1}^{N}{\left|\psi_{j}\left(r,t\right)\right|}^{2}. \end{array}$$

The Kohn–Sham potential $V_{\mathrm{KS}}\left[\rho \right]\left(r,t\right)$, a functional of the electron density ρ(r,t), is given by the following:

(3) $$\begin{array}{c} V_{\mathrm{KS}}\left[\rho \right]\left(r,t\right)=V_{\mathrm{xc}}\left[\rho \right]\left(r,t\right)+V_{H}\left[\rho \right]\left(r,t\right)+V_{\mathrm{ne}}\left(r\right)+V_{\mathrm{laser}\;}\left(r,t\right) \end{array}$$

The first term, $V_{\mathrm{xc}}\left[\rho \right]\left(r,t\right)$, represents the exchange-correlation potential, which accounts for nonperturbative many-body effects and is modeled using the generalized gradient approximation (GGA) in the Perdew–Burke–Ernzerhof (PBE) form [48]. The second term, $V_{H}\left[\rho \right]\left(r,t\right)$, corresponds to the Hartree potential, while the third term, $V_{\mathrm{ne}}\left(r\right)$, describes the electron-ion interactions, modeled using norm-conserving Troullier–Martins pseudopotentials [49] in the Kleinman–Bylander parametrization [50]. Lastly, $V_{\mathrm{laser}}\left(r,t\right)$ represents the external potential from the interaction between the molecule and the laser field, expressed as $V_{\mathrm{laser}}\left(r,t\right)=r\cdot E\left(t\right)$, where E(t) is the linearly polarized electric field of the laser.

(4) $$\begin{array}{c} E\left(t\right)=Ef\left(t\right)\cos\left(\omega_{0}t\right){\hat{e}}_{y} \end{array}$$

The linearly polarized electric field has an amplitude of E=0.06a.u. and a wavelength of 800 nm. Its envelope is trapezoidal, with both the rising and falling edges lasting one optical cycle, and the plateau lasting four optical cycles.

The time-dependent Kohn–Sham orbital wavefunctions were propagated in real time on a real-space grid. The propagation method preserves time-reversal symmetry, and we employ the approximated enforced time-reversal symmetry (AETRS) [51] with a time step of 0.08 a.u.

To mitigate unphysical effects arising from the reflection of the electron wave packet at the boundaries, a complex absorption potential (CAP) [52] is used:

(5) $$\begin{array}{c} \begin{array}{c} V_{\mathrm{absorb}\;}\left(r\right)=\left\{\begin{array}{cc} 0, & 0<r<r_{\max}\\ i\eta \sin^{2}\left[\frac{\left(r-r_{\max}\right)\pi }{2L}\right], & r_{\max}<r<r_{\max}+L \end{array}\right. \end{array} \end{array}$$

Here, L=10 a.u. and η=−0.8 a.u. represent the width and height of the absorbing potential, respectively.

The harmonic spectrum is derived from the time-dependent dipole acceleration a(t) [52]:

(6) $$\begin{array}{c} H\left(\omega \right)={\left|\int a\left(t\right)e^{-i\omega t}dt\right|}^{2} \end{array}$$

where $a\left(t\right)=-\int \rho \left(r,t\right)\nabla H_{\mathrm{KS}}d^{3}r$, $\;H_{\mathrm{KS}}=-\frac{1}{2}\nabla^{2}+V_{\mathrm{KS}}\left[\rho \right]\left(r,t\right)$.

## 4. Conclusions

In this work, we systematically investigated the high-order harmonic generation from long-chain polyacetylene-like molecules, particularly focusing on $C_{18}H_{2}$ and $C_{10}H_{2}$, in comparison with the short-chain molecule $C_{2}H_{2}$. By ensuring the same Keldysh parameter γ for different field intensities, we effectively excluded the influence of ionization potential and isolated the role of molecular size in the HHG process. Our results demonstrate that the HHG intensity significantly increases with molecular length, especially under strong linearly polarized fields perpendicular to the molecular axis. Time-frequency analysis and charge variation tracking further revealed that in long-chain molecules, the terminal carbon atoms exhibit pronounced electron localization and charge oscillations, which are strongly correlated with enhanced HHG signals. In contrast, such behavior is absent in short molecules like $C_{2}H_{2}$, indicating a size-dependent emission mechanism. Consequently, we conclude that the spatial extent of a molecule plays a crucial role in enhancing HHG under specific polarization conditions. These findings provide valuable insights for designing molecular systems for efficient attosecond pulse generation and ultrafast light sources.

## Data Availability

The data presented in this study are available on request from the corresponding author.
